# Loop-Mediated Isothermal Amplification Assay for Identification of Five Human *Plasmodium* Species in Malaysia

**DOI:** 10.4269/ajtmh.15-0569

**Published:** 2016-02-03

**Authors:** Yee-Ling Lau, Meng-Yee Lai, Mun-Yik Fong, Jenarun Jelip, Rohela Mahmud

**Affiliations:** Department of Parasitology, Faculty of Medicine, University of Malaya, Kuala Lumpur, Malaysia; Sabah State Health Department, Sabah, Malaysia

## Abstract

The lack of rapid, affordable, and accurate diagnostic tests represents the primary hurdle affecting malaria surveillance in resource- and expertise-limited areas. Loop-mediated isothermal amplification (LAMP) is a sensitive, rapid, and cheap diagnostic method. Five species-specific LAMP assays were developed based on *18S rRNA* gene. Sensitivity and specificity of LAMP results were calculated as compared with microscopic examination and nested polymerase chain reaction. LAMP reactions were highly sensitive with the detection limit of one copy for *Plasmodium vivax*, *Plasmodium falciparum*, and *Plasmodium malariae* and 10 copies for *Plasmodium knowlesi* and *Plasmodium ovale*. LAMP positively detected all human malaria species in all positive samples (*N* = 134; sensitivity = 100%) within 35 minutes. All negative samples were not amplified by LAMP (*N* = 67; specificity = 100%). LAMP successfully detected two samples with very low parasitemia. LAMP may offer a rapid, simple, and reliable test for the diagnosis of malaria in areas where malaria is prevalent.

Malaria is a devastating disease causing approximately 584,000 deaths in 2013,^1^ but diagnosis still remains a challenge.^2^ Microscopic examination of thin or thick smears of peripheral blood remains the gold standard for the last 100 years. However, it is well documented in many areas that this method is time consuming and misdiagnosis is common especially in mixed infection and/or when the parasitemia is low.^3^ Molecular methods such as conventional polymerase chain reaction (PCR) and real-time PCR may represent a better alternative for malaria diagnosis; however, large-scale application of molecular methods for malaria surveillance has not been implemented, largely due to the high cost of the equipment used.

Loop-mediated isothermal amplification (LAMP) is a rapid and cheap molecular method that enables DNA amplification under isothermal conditions. It is capable in the diagnosis of a wide range of microorganisms associated with bacterial, viral, parasitic, and other diseases in the biomedical field.^4^–^7^ Several LAMP methods have also been developed for malaria diagnosis based on *Plasmodium falciparum* histidine-rich protein-2 (*PfHRP2*),^8^ *18S rRNA*,^9^ *Plasmodium berghei* sporozoite protein with MACPF related domain (*PbSPECT2*),^10^ beta tubulin (*β-tubulin*),^11^ *Plasmodium falciparum* gametocyte specific genes (*Pfs*)16 and *Pf*s25,^12^ mitochondrial DNA,^13^ and apical membrane antigen 1 (*AMA-1*).^14^ However, up to date, none of the LAMP assays were designed to detect and differentiate all five human malaria parasites (*Plasmodium falciparum*, *Plasmodium vivax*, *Plasmodium malariae*, *Plasmodium knowlesi*, and *Plasmodium ovale*). In this study, we report the attainment of higher sensitivity of detection of all five human malaria species through the use of LAMP method developed based on *Plasmodium* small subunit ribosomal RNA (18S rRNA). Results from LAMP were compared with microscopic examination and nested PCR.

Patients, who presented with fever associated with symptoms that doctors on duty considered indicative of a malaria infection (e.g., headache) (*N* = 181), and healthy donors (*N* = 20) blood samples were collected from University Malaya Medical Center (UMMC), Kuala Lumpur, Malaysia, and Sabah State Health Department, Sabah, Malaysia. Microscopic examination and immunochromatographic rapid test were performed. This study was approved by the Medical Ethics Committee of UMMC (reference no. 817.18) and Medical Research Ethics Committee (reference no. NMRR-12-949-11328).

*Plasmodium* malaria species were determined by nested PCR targeted at *18S rRNA* gene.^15^,^16^ FIP, BIP, F3, and B3 primers were designed using the Primer-Explorer V3 software (Eiken Chemical Co., Ltd., Tokyo, Japan) based on *18S rRNA* gene sequences of *P. falciparum*, *P. vivax*, *P. malariae*, *P. knowlesi*, and *P. ovale* (GenBank accession nos. AF145334.1, X13926.1, M54897.1, L07560, and AF145337.1, respectively) (Table 1). Loop primers (Loop-F and Loop-B) were designed manually. LAMP was carried out according to the manufacturer's protocol (Loopamp DNA Amplification Kit with Florescent Detection Reagent; Eiken Chemical Co., Ltd.). The amplification was carried out in a Loopamp real-time turbidimeter (LA-320; Teramecs, Co., Ltd., Tochigi, Japan) with reaction mixture, 4 μL DNA template, and species-specific primers. All experiments were performed in duplicate at least two times. Several endpoint assessments were carried out to confirm the positive reaction. First, the reaction tube was irradiated with the ultraviolet (UV) light; solutions with amplicons showed stronger green fluorescence under UV compared with the solutions without amplicons. Second, hydroxynaphthol blue dye (HNB) was used for the colorimetric detection of LAMP reaction.^17^ A positive reaction was observed by visualizing the color change from violet to sky blue. In addition, PCR was performed on 4 μL LAMP products using F3 and B3 primers. The PCR products were then confirmed by DNA sequencing.^14^

For limit of detection, recombinant plasmids encompassing the target region of the *18S rRNA* gene were constructed for all five *Plasmodium* species. The detection limit of LAMP was determined by serially diluted positive control plasmids. The copy number (10^6^ copies to one copy) was plotted against the threshold time.^14^ Specificity of each species-specific LAMP was examined using genomic DNAs of the other human *Plasmodium* species and various nonhuman primate *Plasmodium* species (*Plasmodium coatneyi*, *Plasmodium cynomolgi*, *Plasmodium fragile*, *Plasmodium brasilianum*, and *Plasmodium inui*) (American Type Culture Collection [ATCC], Manassas, VA).

The clinical specificity and sensitivity of LAMP^14^ were determined based on the results of 201 whole blood samples. A composite diagnosis for each sample (two of three tests giving the same result) was created and used as a reference for all three test modalities.^18^

After microscopic analysis, 132 malaria blood samples (30 *P. falciparum*, 39 *P. vivax*, one *P. malariae*, 57 *P. knowlesi*, one *P. ovale*, and four mixed infections samples) were identified as positive for malaria infections and 49 samples were found to be negative for malaria infections (Table 2).

LAMP was found to be optimal with temperature of 65°C for all positive *Plasmodium* species samples were observed. Amplified products were observed as early as 20 minutes, with a maximal sensitivity reached at 35 minutes. LAMP products were observed either with visual inspection, turbidity reading, or by PCR amplification of the LAMP products. An expected band of ∼200 bp was obtained when the specific LAMP product was used as a template for PCR amplification using B3 and F3 primers, whereas no band was observed from samples containing LAMP product of nonspecific *Plasmodium* species or negative control.

The detection limit for LAMP was 10 copies for *P. knowlesi* and *P. ovale* and one copy for *P. vivax*, *P. malariae*, and *P. falciparum*, respectively. Whereas, nested PCR had a detection limit of 100 copies for *P. knowlesi*, *P. ovale* and 10 copies for *P. vivax*, *P. malariae*. The high specificity of each LAMP assay was confirmed by using genomic DNA of other human *Plasmodium* species. Outcomes showed that LAMP was not observed with DNAs of other human *Plasmodium* species, and cross-reactivity does not occurred between species-specific assays. The analytical specificity of LAMP was also examined by testing genomic DNA of nonhuman malaria species, *P. coatneyi*, *P. cynomolgi*, *P. fragile*, *P. brasilianum*, and *P. inui*. No cross-reactivity was observed with DNA from other *Plasmodium* species, except for *P. vivax* LAMP. *Plasmodium* *vivax* LAMP cross-reactivity with *P. cynomolgi* and *P. fragile* predicted as 99% of *18S rRNA* gene nucleotide sequence homology was observed using ClustalW2 (http://www.ebi.ac.uk/Tools/msa/clustalw2/) among the three species. To evaluate the clinical sensitivity and specificity of LAMP for detection of all five *Plasmodium* species in human samples, we compared the LAMP method with conventional nested PCR, which target on the *18S rRNA* gene, and microscopic examination. Of the 132 samples tested positive with microscopy (parasitemia ranged from 0.01% to 1.52%), all samples were positive with LAMP and 130 samples were positive with nested PCR (Table 2). Specificity and sensitivity of LAMP for all five *Plasmodium* species were 100%, respectively (Table 3).

Under microscopy, *P. knowlesi* generally resembles *P. malariae*, and the early stage of *P. knowlesi* trophozoites resembles those of *P. falciparum*. Thus, the laboratory misdiagnosis of *P. knowlesi* is common.^19^ In this study, two human patients that were initially diagnosed with either *P. knowlesi* or *P. malariae* infection by microscopic examination, presented positive for *P. malariae* and *P. knowlesi*, respectively, by LAMP, whereas nested PCR amplification was unable to detect both of these cases because of very low parasitemia (< 0.01%). These results also showed that LAMP could be useful as confirmatory test of malaria infection especially in differentiation of *P. malariae* or *P. knowlesi* infection, which could not be easily differentiated under microscope because of morphological similarity.

To our knowledge, this study is the first report of using LAMP for the diagnosis of all the five human malaria species. In this study, *Plasmodium* species DNA was successfully amplified within 35 minutes for all five malaria parasites (*P. falciparum*, *P. knowlesi*, *P. vivax*, *P. malariae*, and *P. ovale*) at 65°C, and the sensitivity of LAMP was 10 times higher compared with nested PCR. All species-specific LAMP assays identified all five human malaria species specifically. In addition, we used HNB dye for the colorimetric detection of the LAMP reaction. The benefit of this LAMP is that the HNB dye allows the test to be interpreted using the naked eye by observing the color change,^20^ providing a potential rapid point-of-care method for easy detection of malaria with minimal instrumentation. Combine with the high specificity and sensitivity of LAMP to amplify DNA under isothermal conditions within 35 minutes implies that LAMP may possibly be a good substitute for detection of malaria compared with the nested PCR that requires long operation hour and expensive equipment.

However, there are limitations for LAMP. Because of high sensitivity nature of LAMP, cross-contamination can occur easily. This constraint can be easily overcome by careful pipetting, use of clean bench, and LAMP master mix to avoid frequent exposure of the tube to the environment.

In conclusion, LAMP could be a reliable, convenient, and possible substitute for molecular diagnosis of *Plasmodium* infection particularly in high malaria transmission regions. Its low cost and high simplicity make it a potentially useful tool for point-of-care diagnosis. However, this LAMP assay needs an initial screening for pan-*Plasmodium* detection followed by species identification.

## Figures and Tables

**Table 1:** LAMP primers used in this study

*Plasmodium* species	Primer	Sequence (5′ to 3′)
*Plasmodium knowlesi*	FIP	GTTGTTGCCTTAAACTTCCTTGTGTTCTTGATTGTAAAGCTTCTTAGAGG
	BIP	TGATGTCCTTAGATGAACTAGGCTTTGCAAGCAGCTAAAATCGT
	FLP	TAGACACACATCGTT
	BLP	GCACGCGTGCTACACT
	F3	CCATCTATTTCTTTTTTGCGTATG
	B3	CAGTGGAGGAAAAGTACGAA
*Plasmodium ovale*	FIP	ACCTAGTCGGCATAGTTTATGGTTATTAATCAAGAACGAAAGTTAAGGGA
	BIP	CCTTTCGGGGAAATTTCTTAGATTGTCCCAGAACCCAAAGACTT
	FLP	CGGTATCTGATCGTCTTCAC
	BLP	CTTCCTTCAGTACCTTATGAGA
	F3	GCATTTGCCTAAAATACTTCCAT
	B3	TCAATTCTTTTAACTTTCTCGCT
*Plasmodium vivax*	FIP	GCCATGTTAGGCCAATACCCTAATGTGTGTATCAATCGAGTTTCT
	BIP	TAACGGGGAATTAGAGTTCGATTCCTGTAATTTACGCGCCTGCT
	FLP	CATCAAAAGCTGATAGGTC
	BLP	GGAGAGGGAGCCTGAGAAATAGC
	F3	AGCGACACGTAATGGATC
	B3	CTTGTCACTACCTCTCTTCT
*Plasmodium falciparum*	FIP	AGTAGTCCGTCTCCAGAAAATCTTACTTTGGGGGCATTCGTATT
	BIP	GCGAAAGCATTTGCCTAATCTATTTAAGATTACGACGGTATCTGATC
	FLP	TCACCTCTGACATCTG
	BLP	GTTAAGGGAGTGAAGACG
	F3	GCTTAGTTACGATTAATAGGAGTA
	B3	AGTCGGCATAGTTTATGGT
*Plasmodium malariae*	FIP	GCTTTCGCAGTTGCTTGTCTC-GGGGGCATTTGTATTCAGA
	BIP	ACGAAAGTTAAGGGAGTGAAGAC-AGTCGGCATAGTTTATGGTT
	FLP	CTAAGAATTTCACCTCTGAC
	BLP	GATCAGATACCGTCGTAATC
	F3	AGTTACGATTAATAGGAGTAGCT
	B3	TTACACTATCATCCAACACCT

LAMP = loop-mediated isothermal amplification.

**Table 2:** Results of microscopy, nested PCR, and LAMP assay for detection of all *Plasmodium* species from patients and healthy donors samples

Samples		Microscopy	Nested PCR	LAMP
*Plasmodium falciparum*	Positive	30	29 + 1†	30 + 1†
	Negative	0	1	0
*Plasmodium vivax*	Positive	39	39	39
	Negative	0	0	0
*Plasmodium knowlesi*	Positive	57	56 + 2* + 1†	57 + 1†
	Negative	0	1	0
*Plasmodium malariae*	Positive	1	1	1
	Negative	0	0	0
*Plasmodium ovale*	Positive	1	1	1
	Negative	0	0	0
*P. knowlesi*/*P. vivax*	Positive	2	2	2
	Negative	0	0	0
*P. knowlesi*/*P. malariae*	Positive	2	0	2
	Negative	0	0	0
Microscopy negative	Positive	0	0	0
	Negative	69	67	67

LAMP = loop-mediated isothermal amplification; PCR = polymerase chain reaction.

* Two samples with 0.01% parasitemia (*P. knowlesi*/*P. malariae*) were detected as *P. knowlesi* only by nested PCR.

† Microscopy negative samples were detected positive by LAMP and nested PCR. Parasitemia ranged from 0.01% to 1.52%.

**Table 3:** Sensitivity and specificity of LAMP, microscopy, and nested PCR for all five human malaria species detection

*Plasmodium* species	Method	Specificity (%)	Sensitivity (%)
*Plasmodium falciparum*	LAMP	100	100
	Microscopy	100	96.7
	Nested PCR	100	96.7
*Plasmodium vivax*	LAMP	100	100
	Microscopy	100	100
	Nested PCR	100	100
*Plasmodium knowlesi*	LAMP	100	100
	Microscopy	100	98.2
	Nested PCR	100	98.2
*Plasmodium malariae*	LAMP	100	100
	Microscopy	100	100
	Nested PCR	100	100
*Plasmodium ovale*	LAMP	100	100
	Microscopy	100	100
	Nested PCR	100	100
Mixed infection	LAMP	100	100
	Microscopy	100	100
	Nested PCR	100	50

LAMP = loop-mediated isothermal amplification; PCR = polymerase chain reaction.

A composite diagnosis for each sample based on two out of three methods giving the same result was used as reference.
